# Competitive sport after SARS-CoV-2 infection in children

**DOI:** 10.1186/s13052-021-01166-6

**Published:** 2021-11-06

**Authors:** Giulia Cafiero, Flaminia Passi, Francesca Ippolita Calo’ Carducci, Federica Gentili, Ugo Giordano, Chiara Perri, Melania Hashem Said, Attililo Turchetta

**Affiliations:** 1 Department of Cardiac Surgery, Cardiology and Heart Lung Transplant, Bambino Gesù Children’s Hospital, IRCCS, L.go S. Onofrio 4, 00165 Rome, Italy; 2 Academic Pediatric Department, Immunological and Infectious Disease Unit, Bambino Gesù Children’s Hospital, IRCCS, Rome, Italy

**Keywords:** SARS-Cov-2, Cardiorespiratory assessment, Children

## Abstract

**Background:**

With the gradual resumption of sports activities after the lock-down period for coronavirus pandemic, a new problem is emerging: Allow all athletes to be able to return to compete after SARS-CoV-2 infection in total safety. Several protocols have been proposed for healed athletes but all of them have been formulated for the adult population. The aim of the present study is to evaluate the adequacy of Italian practical recommendations for return-to-paly, in order to exclude cardiorespiratory complications due to COVID-19 in children and adolescents.

**Methods:**

Between April 2020 and January 2021 the Italian Sports Medical Federation formulated cardiorespiratory protocols to be applied to athletes recovered from SARS-CoV-2 infection. The protocols take into account the severity of the infection. Protocols include lung function tests, cardiopulmonary exercise test, echocardiographic evaluation, blood chemistry tests.

**Results:**

From September 2020 to February 2021, 45 children and adolescents (aged from 9 to 18 years; male = 26) with previous SARS-CoV-2 infection were evaluated according to the protocols in force for adult. 55.5% of the subjects (*N* = 25) reported an asymptomatic infection; 44.5% reported a mild symptomatic infection. Results of lung function test have exceeded the limit of 80% of the theoretical value in all patients. The cardiorespiratory capacity of all patients was within normal limits (average value of maximal oxigen uptake 41 ml/kg/min). No arrhythmic events or reduction in the ejection fraction were highlighted.

**Conclusion:**

The data obtained showed that, in the pediatric population, mild coronavirus infection does not cause cardiorespiratory complications in the short and medium term. Return to play after Coronavirus infection seems to be safe but it will be necessary to continue with the data analysis in order to modulate and optimize the protocols especially in the pediatric field.

## Introduction

With the outbreak of the pandemic caused by SARS-CoV-2 in December 2019, global attention was immediately focused on the impact of the virus on the adult population. The epidemiological characteristics have led to think that the pediatric population may be less susceptible to coronavirus. The social distancing policies, strongly imposed in many countries, have significantly contributed to containing the spread of the virus. In particular, the interruption of school, sports and recreational activities have made it possible to protect children and adolescents from exposure to the virus.

The national data relating to the spread of the virus on the Italian national territory indicated, in June 2020, an incidence of 0.9% in the 0–9 age group and 1.6% in the 10–19 age group [1]. The progressive easing of social distancing measures has led to a progressive increase in the incidence of infection in the pediatric population over the following months (4% in the 0–9 years range; 8.4% in the 10–19 years range) with values ​​in any case consistently lower than adults [2].

**Fig. 1:**
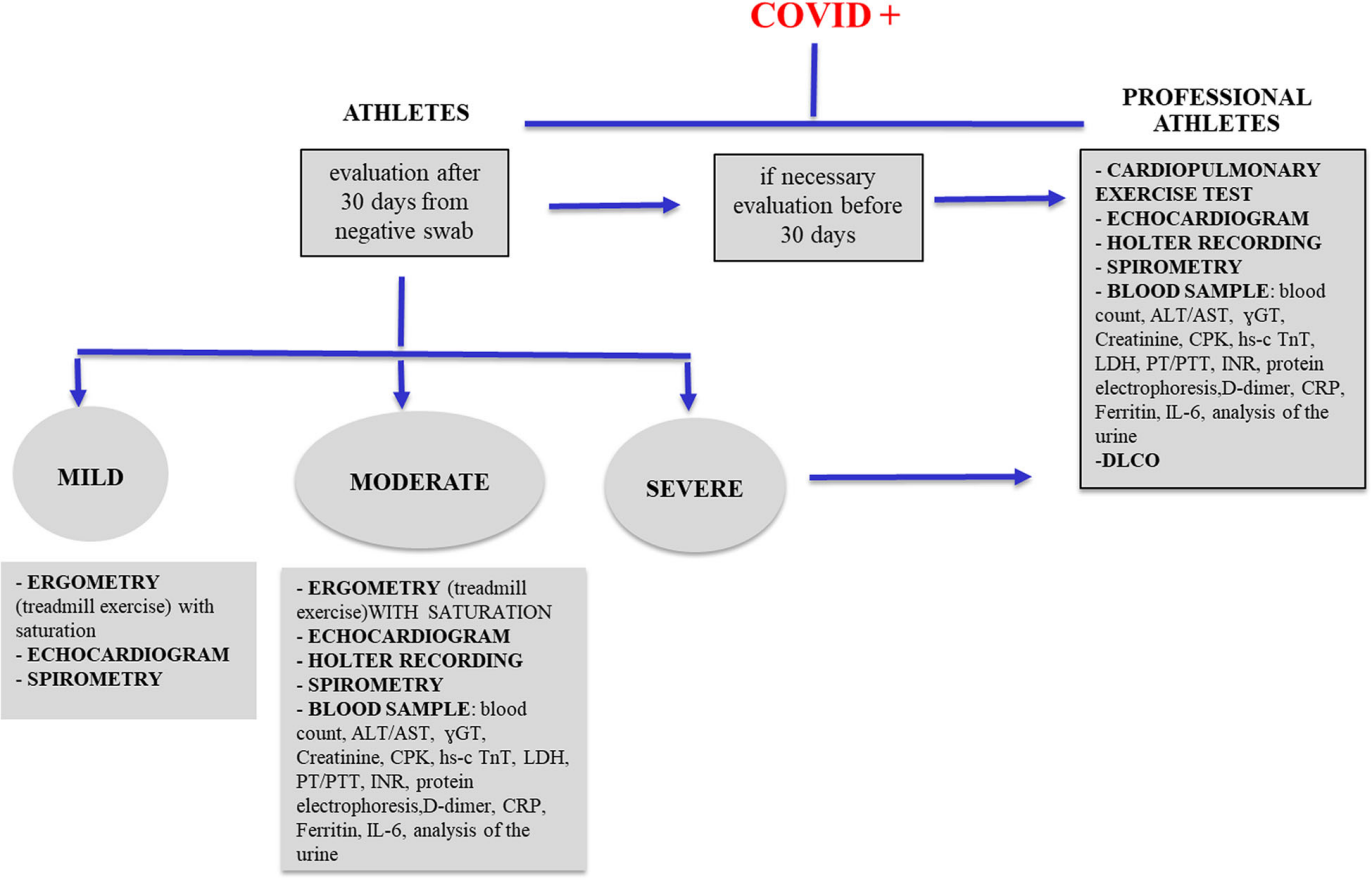
return to play protocol after COVID infection

Despite the increased spread of the virus among the youngest, the prognosis continues to remain significantly better than in the adult population, with a significantly higher rate of asymptomatic or mild symptomatic infections compared to the older age groups [3]. However, it is known that patients with COVID-19 disease may present cardiac involvement with a broad spectrum of clinical manifestations (due to the presence of angiotensin-converting enzyme 2 receptors) [4]. In the literature, the presence of myocardial damage is also described in the absence of symptoms, and cardiac complications can include heart failure, cardiac arrhythmias and sudden death [5]. These evidences have pushed the formulation of comprehensive strategies to ensure a safe return to training and competition for all the athletes [6]. So far there are few data concerning pediatric population; available protocols for a safe return to play concern only adults. In April 2020, in compliance with the regulations for national health prevention [7], protocols dedicated to professional athletes (PA) [8] were published and subsequently reworked to be applied to all athletes (A) engaged in sports at competitive level [9]. (Fig. 1).

The purpose of this study is to evaluate the application of Italian cardiopulmonary protocols for return to competitive sport after SARS-CoV-2 infection to a pediatric population.

## Methods

### Study desing

From October 2020 to February 2021, pediatric patients recovered from coronavirus infection were evaluated according to the protocols in force: the first 17 control patients were evaluated according the PA protocol; the next 28 patients were evaluated according to the revised protocols applied to all athletes (A), not just professionals.

Inclusion criteria: age less than 18 years; previous SARS-CoV-2 infection documented by polymerase chain reaction on a nasopharyngeal swab; negativity for at least 30 days; patients practicing competitive sports.

Exclusion criteria: non-cooperating patients for functional tests due to age and/or psycho-physical limitations; patients with a negative swab for less than 30 days; symptomatic patients (cough, cold, fever); patients with a history of congenital heart disease even if corrected and cured; patients with a positive history of hyper or hypokinetic arrhythmias; patients with bronchial asthma.

It was also decided to exclude one patient with Multi-System Inflammatory Syndrome in Children (MIS-C) as this case was included in a different study protocol.

For all patients we described demographic data, duration of infection, severity of symptoms according to NIH classification [10].

All patients were subjected to a serological test to rule out any false positives.

Blood chemistry tests, including markers of myocardial damage, were performed only in PA group.

All patients underwent an echocardiographic evaluation. Tissue Doppler measurements include both systolic and diastolic measurements (ejection fraction EF%, early diastolic mitral annulus velocity E’, late diastolic velocity A’). Derived measures are the E’/A’ ratio describing diastolic function and conventional E to tissue Doppler E’ ratio (E/E’ ratio), a surrogate of left ventricular filling pressure [11].

Patients in PA protocol underwent a symptom-limited cardiopulmonary exercise test (CPET) using a treadmill, following the modified Bruce protocol with continuous 12-lead electrocardiographic monitoring system.

Data collected during cardiopulmonary test included: respiratory quotient (RQ), the peak of VO2 consumption during the test (Peak VO2, defined as the average value in the last 20 s of the effort in relative –ml/kg/min-values) and the ratio between ventilation and exhaled carbon dioxide (VE/VCO2). We considered the test as maximal when subjects reached at least RQ > 1 and we used for VO2max and VE/VCO2 ratio the cut-off suggested by Takken et al. [12]: a VO2max > 50 mL/kg/min has been considered as normal, 40–50 mL/kg/min good, 20–40 reduced; 35 was considered the cut-off for VE/VCO2 ratio. The strict sanification procedures imposed to limit as much as possible the risk of spreading the infection from SARS-Cov-2 have involved the supply of materials available in particular of facial masks. We decided to use single-use mouthpiece. To be sure of obtaining reliable data, we performed a training period of 1 min of running with the mouthpiece and nose properly closed before carrying out the final test. An operator dedicated to supervision was also provided to help patients, especially the youngest ones, by supporting the mouthpieces at an adequate height and at the same time following the movements during the test. The recording of the parameters was set every two seconds and conducted for at least 1 min before the interruption of the test and 30 s of recovery. Significant and transient changes in the parameters in question during the effort were considered an index of air leak and the tests deemed invalid for statistical purposes. A standard 24 h Holter ECG monitoring was performed in this group.

Patients in A protocol underwent a maximal exercise test using a treadmill, following the modified Bruce protocol with continuous 12-lead electrocardiographic monitoring system.

We considered the test as maximal when the heart rate reached at least 85% of the theoretical value for age.

Desaturation during effort was considered as losing 4 or more point of blood oxygen saturation.

Systolic blood pressure was also obtained at baseline (BP), during and at the end of exercise (BP max).

Lung function was measured by conventional spirometry; Forced Vital Capacity (FVC) and Forced Expiratory Volume at the 1st second (FEV1) were expressed as percentage of predicted values [13].

In the PA group, although not required by the protocols, lung diffusion capacity for carbon monoxide (DLCO) was also assessed, measured by means of the single-breath test. The hemoglobin value was taken for correcting the DLCO. Measurements were expressed as percentages of predicted normal values. Diffusion deficit was considered as DLCO < 80% of predicted value.

All pulmonary function test was performed according to the protocols for the prevention of coronavirus disease [14].

### Data analysis

Continuous variables were described using mean with standard deviation (SD) and compared with unpaired Student’s t-test, if normally distributed, or with Mann-Whitney U test, if not normally distributed. Categorical variables were reported as frequencies and compared with Chi-square test.

A *p* value of < 0.05 was considered statistically significant. All statistical analyses were performed using MedCalc Statistical Software version 15.8 (MedCalc Software bvba, Ostend, Belgium; https://www.medcalc.org; 2015).

## Results

### Patients characteristics

A total of 45 patients were enrolled: 17 in PA group (M = 13); 28 in A group (M = 13).

The mean age of the patients analyzed was 13.97 + 1.9 years.

Data on negative swab times and data on symptoms were collected retrospectively during clinical evaluation: the mean negative time for nasal swabs was 18 + 2.7 days; 25 subjects reported being asymptomatic; 14 subjects reported fever lasting no more than 4 days; anosmia was reported in 6 cases. No case of “long covid” was found. The average time taken by patients to undergo the tests compared to the days of negative swab was 70 + 45 days.

### Functional data

Complete data of the analyzed population are reported in Table 1.

**Table 1:** Functional parameters

	RESULTS
**FEV 1 %**	97,9 (11,5)
**FVC %**	94,6 (12,4)
**VO2/MAX** (ml/kg/min)	41,6 (7,8)
**VE/VCO2**	29,5 (4,7)
**RQ**	1,06 (0,1)
**DLCO %**	91,5 (15,7)
**EF%**	65,9 (4,2)
**E/A**	2,16 (0,6)
**E/e’**	6,21 (0,9)
**DIASTOLIC DYSFUNCTION (n)**	0

Data expressed as mean (standard deviation) except for diastolic dysfunction (n).

FVC = forced vital capacity; FEV1 = Forced Expiratory Volume in the 1st second; RQ = respiratory quotient; DLCO = diffusion capacity of the lung for carbon monoxide; EF = ejection fraction.

At cardiovascular level, all patients obtained results that could be correlated with the current state of training and no new case of arrhythmias were found.

On patient showed arterial hypertension. Before being able to correlate the hypertension to the infection, the patient is carrying out the study to exclude secondary causes.

All the examinations to assess pulmonary function were found to be within the normal limits.

No case of systolic neither diastolic dysfunction has been highlighted.

### Blood parameters

All blood chemistry tests, including markers of myocardial damage, were found within the reference values.

## Discussion

The SARS-CoV-2 pandemic has profoundly affected all aspects of daily life and has not spared any age group. While in the first wave the severe restrictive measures meant that the youngest were just touched by this virus, during the following waves the pediatric-adolescent age saw an increase in the rate of infections. Although this, isolated cardiovascular complications remain very low in children and literature data are scarce or limited to the description of a few isolated cases.

It was therefore essential, before the restart of any activity, including sports, to check the impact of the virus on the health of those recovered from the infection. Several protocols have been formulated to investigate short and medium-term complications on the different organs and systems involved in the infection. The role of ACE-2 receptors in the pathogenesis of the disease is now known [15] and since this receptor is not only present in the lungs but also in the heart, kidney, vascular and intestinal areas, it was essential not to underestimate any theoretical complication. In Italy, since April 2020, strict protocols have been applied for the evaluation of professional athletes before resuming physical activity after coronavirus infection. As the months went by and the gradual reopening, the protocols have been revised to be suitable for the entire population of athletes practicing competitive sports. There are currently no age limits to the application of the protocols. Young people necessarily also fall into these categories. The data collected in recent months at our institute on a pediatric population (aged 9 to 18 years) made it possible to assess the impact of SARS-CoV-2 infection mainly at respiratory and cardiovascular level. As shown by the results, most patients had an asymptomatic or mild symptoms infection. At the end of our observation period in February 2021, only 1one subject was diagnosed with MIS-C. Having subsequently noted a slight increase in MIS-C cases, it was therefore preferred to exclude this patient and enroll it in a dedicated study currently underway. No short or medium term complications were observed, either cardiovascular or respiratory. As evidenced by Ludvigsson et al. [16], almost nothing has been described in the pediatric field about “long covid” and our data does not allow us to offer any conclusions on this complication in children. This study, although preliminary and collected on a selected pediatric population, do not differ from the data currently available in the literature [17]. In relation to the impact of the pandemic on global public health, it was essential to apply the protocols in force before the resumption of sporting activities at a competitive level.

## CONLUSION

The results of the present study make us state that, in the case of mild coronavirus infection, pediatric population is not at risk of developing cardio-respiratory complications even under high physical stress conditions. The main limitation of this study concerns the sample size, however reflecting the incidence of infection in this age group. Another important limit was the difficulty of obtaining a greater number of data from cardiopulmonary tests limited by the long times of sanification of materials. Children and adolescents will continue to be assessed before resuming sporting activity in order to confirm data obtained until now, by progressively including subjects affected by MIS-C or subjects “long covid”. It will therefore be possible to assess the possibility of lightening or even suspending these evaluations in the cases of mild coronavirus infection in the pediatric age groups.

## Data Availability

The data analyzed during the current study are available from the corresponding author on reasonable request.
